# A Conceptual Restructuring of Spatial Motion Expressions in Chinese L2

**DOI:** 10.3389/fpsyg.2018.01698

**Published:** 2018-11-07

**Authors:** Carlotta Sparvoli

**Affiliations:** School of Asian Studies, University College Cork, Cork, Ireland

**Keywords:** spatial motion expression, localizers, locative motion events, axial part, Chinese grammar

## Abstract

This paper focuses on the patterns in the encoding of spatial motion events that play a major role in the acquisition of these type of expressions. The goal is to single out the semantic contribution of the linguistic items which surface in Chinese locative constructions. In this way, we intend to provide learners with an account of the spatial representation encoded in the Chinese language. In fact, Chinese grammar is often perceived as idiosyncratic, thus generating a frustration that turns into learned helplessness (Maier and Seligman, 1976). We will analyze Talmy (2000a,b) framework under the light of investigations such as Landau and Jackendoff (1993), Svenonius (2004, 2006, 2007), and Terzi (2010). It will be shown that in Chinese locative structures, the Axial Part information is signaled by localizers and can be specified only when the Ground is considered as an object with “axially determined parts” (Landau and Jackendoff, 1993). Thus, we will elaborate on present account on the localizer’s function (Peyraube, 2003; Lamarre, 2007; Lin, 2013) by showing that the localizer highlights an axially determined part within a reference object, consistently with Terzi (2010) definition of Place, and with Wu (2015) decomposition of Place into Ground and Axial Part. Moreover, it will be shown that the preposition *zài* ‘at’ encodes a Locative type of Motion event (Talmy, 2000b), thus, it is not semantically vacuous. Other categories will be presented, such as the semantic class of locational verbs (Huang, 1987). We will indicate the contexts wherein such notions can trigger the conceptual restructuring which enables adult learners to switch from L1 “thinking for speaking” to L2 “thinking for speaking” (Slobin, 1987). The paper is structured as follows: Section “Introduction” provides introduction to the theme; Section “Theoretical Framework” includes a surveys on the semantic and syntactic decompositions of spatial motion expressions; Section “Discussion” offers an account of the instantiation; the findings and the relevant pedagogical implications are presented in Section “Findings.”

## Introduction

This paper focuses on expressions of locative and motion events. This type of utterances, while relying on a reference system that anchors a Figure (the moving or virtually moving entity) to a Ground (perceived as a stable point of reference), also tends to leave unspecified contents that can be easily inferred by the hearer. Therefore, L2 learners need to be aided in the understanding of the spatial mental representation adopted in the target language.

The underlying hypothesis is that in the linguistic expression there is a process of “thinking for speaking” in which “one fits one’s thoughts into available linguistic forms” and which “involves picking those characteristics that (a) fit some conceptualization of the event, and (b) are readily encodable in the language” (Slobin, 1987, p. 435). As underlined by Romagnoli (Forthcoming), this hypothesis triggered vast research on the typological variation in the motion event encoding by L2 learners, typically focusing on the difference in the path expression.^1^ This study instead is aimed at shedding light on the semantic contribution instantiated in the Chinese grammatical encoding of motion events. In this way, we want to bring to the front the concepts that can help L2 learners to restructure their mental representation of this type of contents and “think them” consistently with the target language encoding.

We will focus on constructions in the English–Chinese Interlanguage whose ungrammaticality is not clearly explained in the standard account of locative and motion sentences. For instance, sentences like (1a) do not pose any challenge. Conversely, in (1b) learners typically omit the post-position *shang* ‘on’ attached to the place noun. As we will show in Section “Discussion,” these post-positions, generally called localizers, provide semantic information that in English is typically either expressed by a preposition (1a) or left to the hearer’s inference (1b). An opposite yet similar example occurs wherein the source-path information is necessarily specified in English (by the preposition ‘from’) and left unspecified in Chinese, as in (1c).

**Table:** 

(1)	(a)	*Shū zài*	*shūjià-**shang***.
		Book be.located book.shelf **on**	
		‘The book is **on** the bookshelf.’	

**Table:** 

(b)	*Tā*	*cóng*	*shūjià*-^∗^**(*shang*)**	*ná*
	3sg	from-Path	book.shelf **on**	take
	*xia*	*lai-le*	*yì-ben*	*shū*.
	descend	come-PRF	one-CL	book
	‘He took a book from (**over**) the bookshelf.’			

**Table:** 

(c)	*Lóu-shang*	*zǒu*	*xia*	*lai-le*
	floor-on	walk	descent	come-PRF
	*liãng-ge*	*rén*		
	two-CL	person		
	‘Two men came down **from** upstairs.’			

In other words, (1b) and (1c) exemplify two opposite scenarios: when the Axial Part information is marked in Chinese and unspecified in English and when the Path is marked in English and unspecified in Chinese. These examples result from a different way of organizing the cognitive information related to the locative and motion expressions and therefore require a specific focus, which will be provided in Section “Discussion.”

Other challenges in L2 acquisition can arise due to the alternation of different locative constructions, depending on the discourse structure, as in the sentences with subject-predicate inversion (2).

**Table:** 

(2)	?*Yìxiē*	*liúxuéshēng*	*lái-le*	*wǒmen*	*jiàoshì-li.*
	some	foreign.student	come-PFR	our	classroom-in
	‘Some foreign students came to our classroom.’				
	Intended: *Women jiàoshì-li lái-le yìxiē liúxuéshēng*.				

Similarly, another possible challenge for L2 learners is the choice between two different syntactic structures, namely, locative as an adjunct (3a) or as an argument of the resultative verb, (3b).

**Table:** 

(3)	(a)	*Tā*	***zài***	*Běijīng*	*gōngzuò.*
		3sg	**in**	Beijing	work
		‘He works in Beijing.’			
	(b)	?*Tā*	***zài***	*Běijīng*	*zhù.*
		3sg	**in**	Beijing	live.
		Intended: ‘He lives in Beijing.’ *Tā zhù zài Běijīng.*			

Sentences like (2) and (3b) can surface in the English–Chinese interlanguage and suggests that without a focus on the different locative forms, students are typically left with the idea that Chinese grammar structures are idiosyncratic and impossible to be rationally explained. The following section will therefore present a brief survey on basic notions needed for providing a rationale to Chinese spatial construction.

## Theoretical Framework

### Semantic Decomposition

Let us now start with a brief survey on the cognitive semantic model by Talmy (2000a,b). The Talmyan analysis of spatial motion events is primarily aimed at unpacking the components which are being used in the cognition of the “Facts of Motion.” Such facts surface in two alternative states, either motion or stationary. The expressions of this type of information are based on the recognition of two entities, a moving, or conceivably moving Figure whose location or movement is anchored to a reference point or landmark, Ground. The expression of this type of event is based on a reference system where some information is explicitly expressed; others are conveyed inferentially. Crosslinguistically, there are similar patterns in what is explicitly expressed and what is left to the inferential system. Moreover, Ground and Figure are typically nominal phrases. The greatest labor in this kind of expression is assigned to other linguistic items, typically adpositions, spatial particles and verbs of directed motions, whose specific contribution is subjected to a greater language-specific variance. This is the domain of the expression of the Path. To capture all possible spatial motion schemas, Talmy (2000a, p. 341) conceived Path as the sum of three components, as shown in (4):

(4) [Figure  Motion  {MOVE/BELoc}  Path  (=Vector  +  Conformation + Deictic){path/site} Ground] _Motion event_

As visible in (4), Path can be encoded in two ways, as “path proper” or as site. Moreover, both locative and directional motions are comprised in the same class. In fact, “**Path** is the particular course followed or site occupied by the Figure with respect to the Ground” (Ibid.: 342). Therefore, locatives are a “type of Motion events” (Talmy, 2000b, p. 62). In other words, Motion events can be of two type, a situation containing motion (5a) and the continuation of a stationary location (5b) (Ibid. 25). These two types of Motion events are called directional and locative, as exemplified in (5a) and (5b).

**Table:** 

(5)	(a)	‘The pencil *rolled off* the table.’ (Talmy, 2000b, p. 26)^2^		
	(b)	‘The pencil *lay on* the table.’ (ibid).		
	(c)	*Qiānbǐ*	***zài***	*zhuōzi-**shang***.
		pencil	**be.located**.	table-**on**
		‘The pencil is on the table.’		

(5c) illustrate a Chinese locative expression, wherein the verb *zài* expresses the type of Motion event (locative) and the post-position *shang* expresses the conformational portion of information represented in (4) i.e., *site*. This piece of information would be sufficient to account for the semantic contribution of Chinese motion verbs, pre-positions and post-positions. However, in order to trace the way in which spatial motion events are encoded in Chinese, we still need to satisfy two requirements. Firstly, look for an account which better captures the semantics of the so-called “localizers,” and secondly, map Talmy’s terminology with the one which is currently adopted in the semantic and syntactical literature. Therefore, we need to proceed with a survey in which, from time to time, we will clarify the different reading of technical terms such as Place and Axial Part.

### Path as Opposed to Place

The conceptual category of Path was already found in Jackendoff (1983), where Path is the trajectory opposed to Place (the location). In this model, and its subsequent extensions and refinements, locative expressions are opposed to directional expressions. The former has a directional value and is linked to Source, Goal, and Route (*from, to, across* etc.); the latter instead is a function of the Locative thematic argument (*in, at*). From the syntactic point of view, the function Path dominates the function Place (location, or stative as opposed to motion), and a relative hierarchy is also available within the directional component, where the Source Path is dominating the Goal Source (Pantcheva, 2010).

As underlined by Pancheva, the Source>Goal>Place hierarchy is also confirmed by Zwarts (2005) compositional analysis. In the latter, the Source embeds the location (Place) of the starting point (p0), whereas Goal includes the location of the endpoint of the path (p1). The route prepositions typically denote paths wherein the Figure is not located in p(0) and neither in p(1), it is instead in an intermediate point p(i) between the two (Zwarts, 2005, pp 760, 764).

### Locative Type of Motion Events

In Zwarts’ compositional analysis, locatives are the prepositional counterpart of states in the verbal domain, while Goal/Source/Route are the counterpart of the dynamic verbal domain (Zwarts, 2005, p. 742). A similar construal is also expressed by Nam (2012, p. 471): “Stative Locatives denote a place where an event takes place without location change.” It is even more explicitly defined by positing the geometrical idea of *“null-motion* [which is] determined by letting all of (0,1), corresponding to a single point *p*″, as proposed by the mathematician Hassler Whitney (1933, p. 250). Along these lines, it could be argued that locatives denote a type of motion in which the starting point (p0), the ending point (p1) and, obviously, also their intermediate point (pi), coincide. Therefore, they denote that no motion event took place, as the “locative type of motion events” described by Talmy (2000b, p. 62) for the Spanish *estar*. Section “The semantic function of zài” will discuss this issue with reference to the Chinese locative preposition *zài* ‘at.’ Now instead we will discuss the elaboration of the Conformation component in (4), which will help to understand the semantic contribution of the Chinese localizers.

### From Axial Parts to Prepositions

Talmy’s categories are lexicalized by items whose syntactic classification varies based on language-specific factors. In English, the Vector component is specified by prepositions like *from/to/along/at*, whereas *inside/under/above* provide the information referred to the morphology of the Ground, that is, the conformational portion. A reference to the geometrical morphology of the Ground is also found in Landau and Jackendoff (1993). They pointed out an asymmetry in object representation. In recognition and categorization (when objects are conceived as a “what”), detailed geometrical features are represented. However, when spatial relations are at issue, the representation of the object as a “where” (either Ground or Figure) relies on very coarse geometric properties, primarily its main axes. They observe that in spatial cognition, the reference object is conceived in a threefold pattern: as a surface-type, a volume-type, or a line (Ibid. 232). Prepositions such as *in front*, *on top* refer to the object’s axially determined parts, also called ‘axial parts,’ as in the classic definition of this concept which has been provided by Jackendoff (1996, p. 14).

By referring to the Ground’s axial parts, the prepositions describe the region where the Figure is located, that is Place. To describe the routes from place to place, our representation invokes a further mental element, that is Path (Landau and Jackendoff, 1993). Drawing on the Place descriptions, “region operators” are added (*via, to, toward, from, away*) which also specify where the path begins and ends (Ibid. 232).

Importantly, in Chinese such “region operators” are all prepositions, whereas the axial parts information is provided by other adpositions, called localizers. Also, it must be underlined that, under such analysis, the Ground is primarily an object; an object conceived as “where” (as opposed to an object conceived as “what”). We will go back to this issue in Section “Object as Where”. Now we briefly present the most relevant formal analysis of spatial expressions.

### Vector Space Semantics

Building on the idea of axial parts, Svenonius (2004, 2006) proposes a formal adaptation in which spatial prepositional phrases (PlaceP or Loc) denote a vector space. For instance, the vectors which project ‘backward’ are lexically realized by the item *behind.* In this way, the object spatial description is conceived as a rich combinatorial system where we have a further decomposition of Place into two subcomponents: Place and AxPart (axial part). Place specifies “how a vector space is projected from a region, as for *in* and *on* which indicate, respectively, that the space is bounded and that there is contact between Ground and Figure” (Svenonius, 2006, p. 53). AxPart instead identifies a region (a set of contiguous points in space) based on the Ground element. It “translates semantically as a region on the basis of which a vector space is constructed” (Svenonius, 2006, p. 74). It is a function from the region occupied by an object to its subparts, such as *front*, *back*, *top*, *side*, *interior*, or *exterior*. Svenonius outlines a categorial hierarchy wherein, the Place head is lower than the Path head (Svenonius, 2004, p. 9, 2006, p. 59), as visible in (6).

**Table:** 

(6) ‘from in front of the house.’	(Svenonius, 2007, p. 1)


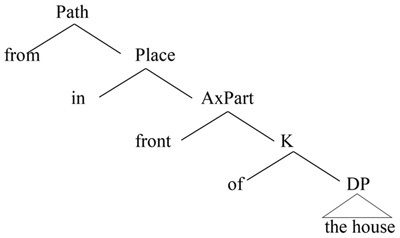


A spatial phrase is here analyzed as a function from the Ground (an object) to an axially determined region. Each semantic component corresponds to a syntactic projection. In a cartographic representation, this is equal to the structure visible in (7), where we have a DP place projection (headed by Place) selected by an overt or covert stative P (Place), whose projection is in turn selected by an overt or covert directional P (Path).

**Table:** 

(7) [P_Dir_ [P_Stat_ [P_AxialPart_ [P [DP]]]]	(Cinque, 2010, p. 7)

Thus, ‘(It was extracted) from under the table’ is derived as follows:

(8) [_PPdir_ from [_PPstat_ AT [_DPplace_ [_AxialPartP_ under X^*o*^ [_PP_P [_NPplace_ the table [PLACE]]]]]]]            (Ibid.)

Therefore, we obtained a more refined hierarchy of the semantic components, as visible in (9), where Place refers to locative as opposed to directional:

(9) Goal>Source>Place>AxialPart>Ground.

### Chinese Prepositional Phrases

Another puzzle in the analysis of spatial constructions pertains to the status, lexical or functional, of spatial prepositions. In this regard, in Terzi’s (2010) analysis there is a shift in the understanding of the notion of Place, which here “denotes the physical space surrounding the reference landmark (i.e., what is considered the Ground argument of the locative)” (Terzi, 2010, p. 196). Locative markers are modifiers of a non-phonologically realized noun (Place) which gives them a “nominal flavor.” The DP containing Place is the complement of a functional head, P_LOC_, which contributes to “their overall oscillating status along the functional/lexical dimension” (Ibid. 196). Building on this type of analysis, and based on Mandarin data, Wu (2015, p. 223) proposes the structure visible in (10). The spatial prepositional phrases are here composed of an articulated configuration with the presence of a phonologically null Place noun merged with the Axial Part phrase below the PLoc(ative) projection.

(10)

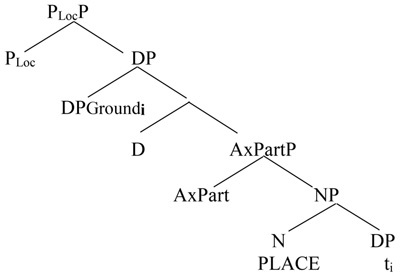

(Wu, 2015, p. 223)

The main syntactic difference between spatial prepositions and adpositions is that spatial prepositions can check the case, whereas the post-positions select DP arguments, and receive their case from the preposition or from the closest verbal projections (as in the directional complement).

Wu’s analysis in turn explains the ability of post-positions, but not preposition, to occur in positions where case is checked, such as a variety of subject positions (as we will see section “Fronted Locative Constructions”).

The categorial syntactical categorization of the so-called localizers has been a matter of debate. There are three main accounts: as post-position, noun, or clitic (Wu, 2015, p. 211ff). Evidence on the adpositional status of the monosyllabic localizers (as *shàng* ‘on’) is provided by Paul who therefore refers to the spatial prepositional phrases as circumpositional phrases and labels the disyllabic forms (as *shàngbian* ‘upper side’) as Location nouns (Paul, 2015, p. 93). For Wu (2015), the post-positions (the localizers) are adpositional projections whose features are mitigated by a covert Place noun merged within them. The monosyllabic forms are unambiguously adpositional elements, the disyllabic ones shift ambiguously between adpositions and nouns (Wu, 2015, p. 219).

Here, we will focus on the main converging elements between these two models. Namely, the default function of spatial prepositions is Path (Paul, 2015, p. 129) and all them (whether directional or locative) compete for the same syntactic position (Wu, 2015, p. 224). Post-positions instead denote what Svenonius (2006, 2007) has labeled as Axial Part information. The crucial point is that “In Mandarin the P_LOC_ and AxPart heads are spelled out by separate/free-standing lexical items” (Wu, 2015, p. 227). The presence of overtly separate adpositional heads shows how Chinese often decomposes categorical distinctions into different syntactic heads for features that in English are typically “condensed” into one head/element, thus confirming the analyticity of Chinese (Huang, 2005), as opposed to a synthetic language such as English.

### The Semantic Function of *Zài*

Now we can turn back to the issue of the semantic contribution of Chinese locative prepositions. If we assume that Chinese spatial prepositions express Path, then we need to address the following questions:

(a) Why does the Chinese inventory of spatial prepositions include an item which is a function of the “Place thematic argument,” i.e., *zài* ‘in/at’?
(b) What is the semantic contribution of *zài*? In most cases, it seems to be a redundant specification of what is expressed by the localizer. For instance, in (11), *zài* ‘at’ on the one hand cannot be omitted, on the other it does not seem to express anything in addition to what *lĭ* ‘inside’ already expresses.

**Table:** 

(11)	(a)	*Tā*	***zài***	*píbāo-**li***	*fàng-le*
		3SG	**at**	handbag-**inside**	put-PERF
		*tài*	*duō*	*dōngxi.*	(Paul, 2015, p. 123)
		too	much	thing	
		‘He put too many things **in** the handbag.’			

**Table:** 

(b)	*Háizi*	***zài***	*wūzi-**li***	*pǎo.*	(Chu, 2009,
	child	**at**	room-**inside**	run	p. 66)
	‘The child is running in the room.’				

Y.-H. Audrey Li argues that the preposition *zài* is responsible for the syntactic Case-assigning function, whereas the semantic function is performed by the localizers (Li, 1990, p. 33), as *lĭ* ‘inside’ in (11). Paul observes that, in Chinese circumpositional phrases “headed by z*à*i ‘at,’ the precise semantics is provided by the Post-P, not by the functional preposition *zài*” (Paul, 2015, p. 125).

From a functionalist perspective, Chu (2009) observes that (11b) includes an explicit mention to a motion event (denoted by *pǎo* ‘run’), but “the sentence expresses no change of location of the Figure” with reference to the Ground. For Chu in (11b) no path is being profiled. Yet, in his very wording, “no change of location” echoes an idea we have seen in Section “Locatives as Type of Motion Events.” Namely, that “Stative Locatives denote a place where an event takes place without location change” (Nam, 2012, p. 471). In sum, the locative can also be interpreted as a sort of null-motion, where no change of place occurs, where the starting point p(0), the ending point p(1), and their intermediate point p(i) coincide.

### Interim Summary

Based on (a) the observations that syntactically all spatial prepositions in Chinese have a default Path function and compete for the same head projections (Paul, 2015; Wu, 2015), (b) Talmy’s understanding of Path as a category comprising both locative and directional contents (which can instantiate as verbs or as prepositions), and (c) based on the semantic analysis by Zwarts (2005) and Nam (2012), we here propose that *zài* denotes a “locative type of motion events.”

As a result, the preposition *zài* is not redundant. More specifically, the localizer expresses the Axial Part [as *lĭ* ‘inside’ in (11a)], whereas *zài* denotes the type Motion event, that is, it signals a null-motion type. In Section “*Zài* in Resultative Construction,” we will produce another piece of evidence on the status of verbal *zài* as a marker of null-motion, but before then we need to briefly discuss the Chinese instantiation of spatial motion events.

## Discussion

### Spatial Motion Events in Chinese

Based on the literature presented in Section “Theoretical Framework,” we can now break down the constituents of spatial motion expressions and observe that Chinese has dedicated markers for each semantic component. This decomposition is of capital importance, because in English prepositional phrases path and axial parts are conflated in the same syntactic category, while in Chinese they are spelled out by different items, prepositions or post-positions respectively. In fact, whereas Ground is crosslinguistically instantiated as a noun, *Path* and *Axial Part* can be encoded by lexical material such as prepositions, adpositions and particles. Concerning Chinese, the spatial constructions typically surface as a tripartite construction, that is, as a circumpositional phrases, with the structure:

**Table:** 

(12)	(a)	**Path**	**Ground**	**Axial Part**
		Preposition	Noun	Post-position
		*Zài*	*zhuōzi*	*shang*
		at	table	on
		‘on the table’		

**Table:** 

(b)	**Path**	**Place**
	Preposition	Disyllabic Localizer/Noun
	*Zài*	*wàimiàn/Bĕijīng*
	at	outside/Beijing
	‘Outside/in Beijing’	

Based on Wu’s syntactical derivation, when Ground is conceived as an axially determined object, the slot of the axial part is filled (13a). When Ground is not axially determined, the axial part information is not provided, therefore the axial part slot is empty (13a), and no “silent localizer” is postulated. Since a locative modifier of NPlace is missing, the physical space of the event remains less precise (Terzi, 2010, p. 196).

**Table:** 

(13)	(a)	[_PLocP_ PLoc-*zài* [_DP_ [_DP–Ground_ *zhuōzi*]_i_ [D [_AxPartP_	
		at	table
		AxPart-*shang* [_NP_ N-PLACE t_DP–Ground–*zhuōzi*_]]]]]	
		on	
	(b)	[_PLocP_ PLoc-*zài* [_DP_ [_DP-Ground_ *Běijīng*]_i_ [D [_AxPartP_	
		at	Beijing
		AxPart-ø [_NP_ N-PLACE t_DP–Ground–*Běijīng*_]]]]]	

The Axial Part is conveyed through localizers (*shang* ‘on,’ *xià* ‘under,’ *lĭ* ‘inside,’ etc.). Based on the structure visible in (10), Place is expressed by the combination of Ground and Axial Part (12a) and can also be conveyed by a disyllabic localizer or by a Place noun (12b). Path trajectory is typically marked by two kinds of items: prepositions expressing Goal, Locative, Source, or Routes (*zài* ‘at,’ *dào* ‘to,’ *cóng* ‘from,’ *xiàng/wăng* ‘toward,’ *yán* ‘along’) and path of motion verbs (*jìn* ‘enter,’ *chū* ‘exit,’ *xià* ‘descend,’ *shàng* ‘ascend,’ etc.). Importantly, the inventory of these three items (localizers, spatial prepositions, and path verbs) includes a number of homophonous items (see Table 1). For instance, *xià* occurs as a localizer (‘under’) and as a Path verb(‘descend’).

**Table 1:** Path and axial part markers in Chinese.

Semantic component	Syntactic class	Main lexical items
Axial part	Monosyllabic post-position (localizers)	Prepausal position, or as isolated morpheme: Lǐ ‘inside,’ *wài* ‘outside,’ *shàng* ‘top,’ *xià* ‘bottom,’ *qián* ‘front of,’ *hòu* ‘back of,’ *nèi* ‘within,’ *zhōng* ‘mid,’ and páng ‘side’. In combination with DP-Ground: unstressed
Path of motion	Prepositions	*dào* ‘to,’ *wãng* ‘toward,’ *xiiàng* ‘toward,’ *cóng* ‘from,’ *yán* ‘along,’ Null-path: *zài* ‘at’
	Verbs	As main verbs: *shàng* ‘ascend,’ *xià* ‘descend,’ *chū* ‘exit,’ *jìn* ‘enter,’ *huí* ‘return,’ *guò* ‘pass,’ *qi(lái)* ‘rise,’ *dào* ‘arrive’ Null-path: *zài* ‘be located’ In resultative compounds: unstressed
Deictic motion	Verbs	As main verbs: *qù* ‘go,’ *lái* ‘come’ In resultative compounds: unstressed

The next sections will instead address the following issue: when is the localizer mandatory (and why)? In this way, we intend to provide an account for the asymmetry exemplified in the examples (1b) and (1c). Firstly, we will resume the most influential understanding of the localizer function. Secondly, we will try to define its role, based on the semantic function of Axial Part which has been assigned to the localizers in a cartographic syntactical approach.

### Locative Expressions

#### The Usage of Localizers

Localizers are defined as morphemes indicating the spatial position of the Figure relative to the Ground NP (Lamarre, 2007, p. 2):

“*Localizers* are unstressed and suffixed on the Ground NP. Apart from their role of marking the NP as a place-word, they indicate the spatial position of the Figure relative to the Ground NP, like 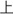 *shang* ‘on,’ or 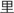 *li* ‘in,’ the two most widely used localizers.”

As underlined by Lamarre (2007), the localizers can be omitted when the Ground is a Place-noun and the spatial relation is that of being *in* the site.

**Table:** 

(14)	*Wǒ*	***zài***	*Běijīng*-(^∗^***li***)	*zhù-le*	*wǔ*	*nián*
	1sg	**in**	Beigjing-**in**	live-PRF	five	year
	‘I have lived **in** Beijing for 5 years.’					

The specific issue of when omitting/adding the localizer has been addressed in detail by Lin (2013). She argues that, when occurring in a spatial-motion schema, a noun like *Beijing* provides the default information that the Figure is within the boundary suggested by the Ground. Therefore, the localizer *li* ‘in’ must not be used, as in (14). Moreover, she points out that:

“a localizer needs to occur and convert the common noun into a place word if the information conveyed in the verb and the physical and functional properties of the Ground is not sufficiently specific to identify the Figure’s location with respect to the Ground at the end of the motion event” (Ibid.: 868).

A minimal pair confirming this claim could be given confronting (1b), here quoted as (15a), with (15b). As observed by Lin (2013), the localizer is not required when Ground follows a Path verb that encodes the same spatial motion event denoted by the localizer, as *shàng* ‘ascend’ with reference to *shàng* ‘top’ (15b), as *jìn* ‘enter’ with reference to *lǐ* ‘inside’ (15d). An opposite behavior is observed when Path expresses a different spatial motion event, as for *dào* ‘arrive’ vs. *xià* ‘under’ (14c).

**Table:** 

(15)	(a)	*Tā*	*cóng*	*shūjià -^∗^(**shang***)	*ná*
		3sg	from	book.shelf-**top**	take
		*xia*	*lai-le*	*yì-běn*	*shū*.
		descend	come-PRF	one-CL	book
		‘He took a book from (**over**) the bookshelf.’			

**Table:** 

(b)	*Mǎyǐ*	*pá*	***shàng***	*zhuōzi*-(^∗^***shang***)	*qu.*
	ant	crawl	***ascend***	table-**top**	go
	(modified from Lin, 2013, p. 865)				
	‘The ant crawled on the table.’				

**Table:** 

(c)	*Mǎyǐ*	*pá*	***dao***	*zhuōzi*-^∗^(***xia***)	*qu.*
ant	crawl	**arrive**	table-**under**	go
	(Ibidem)				
	‘The ant crawled under the table.’				

**Table:** 

(d)	*Tā*	*zǒu*	***jin***	*jiàoshì**-(^∗^li)***	*qu*,
	he	walk	**enter**	classroom-**inside**	go
	‘He went out of the classroom.’				
	(modified from Cai, 2006, p. 68)				

In sum, using the terminology here adopted, Lin (2013)^3^ provides evidence that in Chinese the information about the position of Figure in relation to the geometrical feature of Ground (i.e., Axial part information), is never unspecified. It is morphologically specified by the addition of a localizer. Such a marker can be omitted only when the relevant semantic information is retrievable from the lexical specification of either the Ground or the Path verb.

#### Stereotypical Location

In (13), ‘I have lived in Beijing for 5 years,’ the localizer *lǐ* ‘inside’ is omitted. To capture the difference between the omission and presence of this localizer, it might be useful taking into account an observation by Talmy, on difference between ‘in’ and ‘inside.’

“*Inside* is somewhat more specific than *in.* It seems to require that its reference object be or contain a bounded enclosure (a negative part or the interior of a hollow volume).” Jackendoff and Landau, 1991, p. 155

Landau and Jackendoff (1993, p. 227) also observe that *inside* is appropriate for ‘cave’ and ‘bottle,’ but not for ‘swimming pool or lake, because they are not conceptualized as enclosures or containers.” Also, the same authors underline the difference between “To be at a desk or to be at sink, which signify much more than being located at the desk or at the sink, but rather performing a certain action, such as write or washing” (Landau and Jackendoff, 1993, p. 231). These examples provide an effective parallel for capturing the semantic of another Chinese instance of localizer drop. The localizer *li* is often omitted when the Ground is prototypical locus of the event described by the main verb, as in (16):

**Table:** 

(16)	*Wǒmen*	***zài***	*jiàoshì*	*xuéxí*.
	we	**in**	classroom	study
	‘We study in the classroom.’			

However, the localizer is typically required when the Ground is not semantically related to the VP as its prototypical locus, as in (17).

**Table:** 

(17)	*Tā*	***zài***	*jiàoshì-(****li***)	*zuò*	*fàn.*
	3SG	**at**	classroom-**inside**	make	food
	‘He cooks in class.’				

In sum, a common noun is interpreted as Place noun also based on the event expressed by the main predicate. For instance, in (16) the localizer is preferably omitted because the Ground (‘classroom’) is the prototypical place for the event being expressed (‘study’). However, when the event is not closely semantically related to the Ground, as for ‘classroom’ and ‘cook’ in (17), then the localizer *lǐ* is mandatory. As anticipated in the previous section, when the localizer is omitted, the axial part slot in the syntactic derivation is empty.

#### Noun of Place vs. “Object as Where”

Let’s now discuss the instances in which the localizer is mandatory, even if following a place noun.

In (18a) the localizer follows a modified noun phrase. However, when there is no modifier, the localizer does not occur (18b).

**Table:** 

(18)	(a)	***Zài***	*zhè*	*hēi′àn*	***de***	***Bālí-^∗^(li)***
		**at**	this	obscure	**SUB**		**Paris**-inside	
		*bèi*	*qín*	*shì*	*qīcǎn*	*de*	*shì.*
		BEI	capture	be	miserable	SUB	story
		‘It is a miserable thing to be caught in this dark Paris.’					

**Table:** 

(b)	***Zài***	***Bālí*-(*^∗^li*)**	*bèi*	*qín*	*shì*
	**at**	**Paris-**inside	BEI	capture	be
	*qīcǎn*	*de*	*shì.*		
	miserable	SUB	story		
	‘It is a miserable thing to be caught in this dark Paris.’				
	(PKU Corpus)				

In (19) the modifier *tǒngyī* ‘unified’ suggests that the Ground is here conceived as an object that can be decomposed in different regions, therefore, the occurrence of the localizer is felicitous. In this scenario, the localizer can be stressed. Interestingly, the informants consulted in this study agree on the following: in (18a) the localizer can be unstressed, whereas in (19) it is definitely stressed, arguably for reasons of emphasis.

**Table:** 

(19)	***Zài***	*yí-ge*	*tǒngyī*	***de***	***Ōuzhōu-lǐ***
	**at**	one-CL	unify	**SUB**	**Europe-inside**
	*yě*	*bù*	*yīng*	*yǒu*	*fàxīsīzhǔyì*
	too	nor	should	exist	fascism

**Table:** 

*hé*	*fǎnyóutàizhǔyì*	*de*	*wèizhì.*
and	antisemitism	SUB	position
‘Nor should there be any place for fascism and antisemitism **in** a unified **Europe**.’			
(PKU Corpus)			

In (20) the localizer *nèi* ‘within’ signals that the Figure (the Balkan States) is located within the boundaries of the Ground (Europe) and that it is a part of it.

**Table:** 

(20)	*Tā*	*jiāng*	*tíchū*	*shǐ*	*Kēsuǒwò*	*yǔ*	*qítā*
	3sg	FUT	raise	cause	Kosovo	and	other

**Table:** 

*Bā′ěrgàn*	*guójiā*	*yìdào*	*chéngwéi*	***Ōuzhōu-nèi***
Balkan	nation	together	become	Europe-within

**Table:** 

*miǎnqiānzhèng*	*qū*	*yī-bùfèn*	*de*	*tiáojiàn.*
visa-free	area	one-part	SUB	condition
‘It will set the conditions for Kosovars to join the rest of the Balkan States in the visa-free zone within Europe.’				
(*daccess-ods.un.org*)				

In (21), the localizer follows a Place noun in a modifier position; the Place noun here is considered as an object where a top and bottom (the ‘tail’) position can be identified, therefore the presence of the reference to the axial part is acceptable.

**Table:** 

(21)	*Yīngguó*	*jīnnián*	*shǒu*	*jì*	*GDP*	*jì*
	England	this.year	first	quarter	GPD	quarter

**Table:** 

*zēng*	*jǐn*	*0.2%*,	***zài***	***Ōuzhōu-li***	*de*
increase	only	0.2%	**at**	**Europe-inside**	SUB
*páimíng*	*diàochēwěi.*				
rank	lowest.rank				
‘In the first quarter of this year, the United Kingdom’s GDP rose only 0.2% quarter-on-quarter, its ranking in Europe was the tail of the crane.’					
(*www.chinatimes.com)*					

In (22), once more, the Ground is a Place noun (Brussels) followed by a localizer. The Figure (Woluwe-Saint-Pierre) is a part of it. In this context, the Place noun is considered as an object in which single constituents can be identified. Therefore, the localizer is acceptable.

**Table:** 

(22)	*Wòlǔwéi-Shèng-Píāiěr.qū*,	***zài***	***Bùlǔsài′ěrshì-lǐ***
	Woluwe-Saint-Pierre.area	**at**	**Brussels -inside**

**Table:** 

*suànshì*	*jiào*	*fù*	*de*	*le.*
considered.as	quite	rich	SUB	SFP
‘In Brussels, Woluwe-Saint-Pierre is ranked as a quite wealthy district.’ (PKU Corpus)				

In the examples above, the localizer does not have the function of turning a common noun into a Place noun. It simply provides an information of the position of the Figure, in a context wherein the Ground is analyzed as an object having different axial parts. Importantly, the localizer cannot be dropped when the axial part expresses what Talmy calls “geometrical relations,” as *under*, *above* etc.

**Table:** 

(23)	***Fúdǎo-shang***	*de*	*jūnshìjīdì*	*zúyǐ*
	Fukushima-on	SUB	military.base	be.sufficient
	*wēixi*	*dào*	*zhěnggè*	*Nánměi*.
	threaten	achieve	entire	South.America
	‘The military base in Fukushima was enough to threaten the entire South America.’ (*www.epochtimes.com*)			

Also, Place nouns are compatible with all the localizers expressing absolute reference or an object-centered frame (Levinson, 2004), as ‘north/south’ or ‘next to’, etc. Though it must be underlined that they often occur in disyllabic forms.

**Table:** 

(24)	***Huìzhōu-pángbiān***	*de*	*Xiānggǎng*, *Shēnzhèn*
	**Huizhou-next**	SUB	Hong Kong Shenzhen

**Table:** 

*tèqū*	*hé*	*Guǎngzhōu*	*shì*
special	and	Canton	city
‘The special economic zones of Hong Kong and Shenzhen, and the city of Guangzhou city, next to Huizhou.’			
(PKU Corpus)			

These simple examples allow us to reverse the term of analysis of the role of the localizers. In the literature their function is generally associated to the shift from a common noun to a Place noun (Peyraube, 2003; Sun, 2006; Lamarre, 2007) adding that such transformation is required only when there are no other element suggesting the spatial relation between Figure and Ground (Lin, 2013).

But, if we accept that localizers express the Axial Part, then it must also be assumed that they refer to an “objectified where,” mentally represented via axes which allow us to identify its axially determined parts (Landau and Jackendoff, 1993). This type of decomposition is entirely possible for nouns denoting things, but it is not viable with Place-words. It is, in fact, intuitive, that a Place can be conceived as a deconstructable thing only if we consider it with reference to its component (20, 22), or with reference to a position which requires a higher degree of specificity (24).

### Locatives Phrases as Argument

We can now turn our attention to the behavior of locative phrases as argument in post-verbal and preverbal position, i.e., in directional resultative constructions and locative fronted constructions. Following Huang (1987), in Section “Choosing Between Argument and Adjunct,” we will highlight that there is a specific semantic requirement for these constructions to be allowed, namely the main verbs must be ‘locational.’ Before then, we need to go back to the null-path of motion feature of *zai*, analyzing its behavior when instantiated as a path verb in a directional compound.

#### Zài in Resultative Construction

In the following examples the same lexical material occur either with *zài* ‘be located’, (25a), or *jìn* ‘enter’ (25). In a Directional Resultative Compound, path verbs are unstressed,^4^ and this is confirmed both for (25a) and (25b). However, the former requires the usage of the localizer *lǐ* ‘inside,’ whereas the latter does not.

**Table:** 

(25)	(a)	*Tā*	*bǎ*	*shū*	***fàng***	***zai**-le*	*píbāo-^∗^(**li**)*
		3sg	BA	book	**put**	**be.located**-PRF	bag-**inside**
		‘He put the book into the bag.’						

**Table:** 

(b)	*Tā*	*bǎ*	*shū*	***fàng***	***jin**-le*	*píbāo-(**li**)*
	3sg	BA	book	**put**	**enter**-PRF	bag-**inside**
	‘He put the book into the bag.’					

Consistently with Lin’s “localizer condition” outlined in Section “The Usage of Localizers,” since Path entails a specific spatial interpretation, the NP-PLACE “inherits” it through case assignment, without the need for a localizer to be phonetically realized.

(26) …[_V P_ t_V–*fàng zai*_ [_PLocP_ PLoc-ø [_DP_ [_DP–Ground_ *píbāo*]_i_[D [_AxPartP_ AxPart-*li* [_NP_ N-PLACE t_DP–Ground–píbāo_]]]]]]
  …[_V P_ t_V–*fàngjin*_ [_PLocP_ PLoc-ø [_DP_ [_DP–Ground_ *píbāo*]_i_[D [_AxPartP_ AxPart- ø [_NP_ N-PLACE t_DP–Ground–*píb*ā*o*_]]]]]]

The spatial interpretation of *jìn* is that the figure “crosses a boundary and moves into the enclosed region,” hence the presence of the axial part marker “is not preferred because of information redundancy and only occurs for pragmatic purposes” (Lin, 2013, p. 868). Why then it is required with *zài*? It is then obvious that the spatial interpretation of *zài* ‘be located,’ and that of *jìn* ‘enter’ are not equally related to the notion of being within a given boundary. More specifically, the semantic contribution of the path verb *zài* (similarly to the path preposition *zài*) is not so much that of expressing that “something is located somewhere,” but rather signaling that “something is not moving from somewhere,” consistently with Nam (2012) definition of stative locative (see sections “Locative Types of Motion Events” and “The Semantic Function of Zài”).

#### Choosing Between Argument and Adjunct

Let’s now briefly discuss another scenario which typically puzzles L2 learners, that we have anticipated in (3), here quoted as (28). Namely, the choice between a locative prepositional phrase (26a) and a locative resultative construction (28b). A robust guideline in the choice between prepositional and resultative constructions can be provided by adopting the distinction between locational and non-locational verbs, wherein the former are: “transitive or intransitive verbs that subcategorize for a locative phrase. They include intransitives like *zhù* ‘live,’ *zuò ‘*sit,’ *tǎng* ‘lie,’ *piāo* ‘float’ and transitives like *fàng* ‘put,’ *guà* ‘hang,’ and *xiǎ* ‘write’ (Huang, 1987, p. 228).”

Learners might be interested to know that with locational verbs (mainly posture and displacement verbs) the locative phrase is introduced by a path of motion verb, like *zài*. An appropriate exposition to this type of sentence would be a perfect introduction to the broader class of Manner-Path constructions.

**Table:** 

(28)	(a)	*Tā*	***zài***	*Běijīng*	*gōngzuò.*
		3sg	**in**	Beijing	work
		‘He works in Beijing.’			

**Table:** 

(b)	*Tā*	*zhù*	***zài***	*Běijīng.*
	3sg	live	**in**	Beijing.
	‘He lives in Beijing.’			

Since the choice of the appropriate construction depends on the predicate’s lexical semantics, then learners need to familiarize with the main semantic classes. The latter can be boiled to the three type of motion verbs identified by Talmy (Path, Manner, Deictic), wherein Manner includes the Locational category (posture and displacement verbs), as described by Huang (1987).

Let us analyze the contexts in which the spatial preposition is omitted, thus providing an account for example (2).

#### Fronted Locative Constructions

Locative phrases can be fronted in marked constructions wherein the Place components (Ground + Axial Part) are in the subject position and the preposition conveying the Path information is omitted. These constructions are typically referred to as presentative, existential, and locative inversion sentences, whereas in the Chinese linguistic tradition they are termed as *cūnxiànjù* 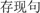.^5^ In locative inversion sentences, the prepositional head gets incorporated onto the governing head (i.e., the verb); as visible in (10), P_LOC_ and AxialPart are spelled by two different head, without overt head movement; the null PLACE gets licensed by the adjacent overt AxPart head so that the phi-features can be accessed (Wu, 2015, p. 225). This explain why in Mandarin, the locative acts like a subject.

A typical environment for these constructions is with an unaccusative verb and indefinite subjects (Huang, 1987). In these cases, the semantic content of the fronted locative phrase can be inferred from the main predicate, as in (1c) “two men came down from upstairs,” whose syntactic derivation is visible below:

(29) [TP [_PLocP_ PLoc-ø [_DP_ [_DP_ [_DP–Ground_ *lóu*]_i_ [D [_AxPartP_
  AxPart-*shang* [_NP_ N-PLACE t_DP–Ground–*lóu*_]]]]]]_i_
  T [_AspP_ t _l*ó*u–shang_ [_Asp′_ [_Asp°_ [_V°_ *zŏu-xia*] [_Asp°_ – *le*]]
  … [_VP_ t_i__*zŏu xia*_ [_DP_ *liăng-ge rén*] t_i_]]

Another typical scenario is with verbs marked with durative aspect *zhe*, typically displacement verbs or posture verb, as in (30a). They do not denote directed motion but a stative locative condition, as for *yǒu* ‘exist,’ *fàngzhe* ‘be located,’ etc.

**Table:** 

(30)	(a)	*Chuáng-**shang***	***tǎng-zhe***	*yí-ge*	*bìngrén.*
		bed-top	lie-DUR	one-CL	patient
		(Huang, 1987, p. 228)			
		‘In the bed lies a patient.’			

**Table:** 

(b)	*Bīngxiāng-**li***	***fàng-zhe***	*yìxiē*	*shuǐguǒ.*
	fridge-**inside**	put-DUR	some	fruit
	‘There is some fruit in the fridge.’			

These constructions can be accounted for with reference to the indefiniteness effect, that is a property in Chinese discourse structure wherein indefinite subjects are accepted in subject position only in specific context.^6^ To understand the inherent logic of sentences like (1c), learners need to become familiar with the mechanism of predicate subject inversion that takes place with indefinite subjects. A focus on this aspect would help the learner to internalize the strategy used in Chinese for conveying definiteness and indefiniteness (which sometimes is strikingly similar to the one adopted in other languages, such as Italian).

The internalization of the verb semantic categories is essential to account for the main locative constructions. Table 2 shows their linear order mapped onto the above-mentioned semantic classes. In particular, four environments can be singled out, wherein, differently from English, the Figure is located in preverbal position (c, d, g, h).

**Table 2:** Main locative constructions in Chinese.

Linear order	Chinese	English
(a) Deictic + Place	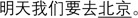	Tomorrow we will go to *Beijing*.
(b) Nul-Path (locative) + Place [canonical]	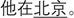	He works in *Beijing*.
(c) Place + Existential verb [loc. inversion]	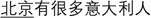	There are many Italians in *Beijing*
(d) PP + Non-locational verb	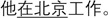	He works in *Beijing*.
(e) Manner + Path + Place	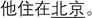	He lives in *Beijing.*
(f) Manner + Path + Place + Deictic	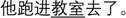	He ran into the *classroom*.
(g) Place + Deictic-*le* + Subject	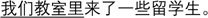	Some foreign students came to our *classroom.*
(h) Place + Locational Verb-*zhe/le* + Subject	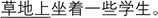	Some students are sitting on the *grass.*

## Findings

In Section “Discussion,” we have shown an alternative account to the claim on the semantic redundancy of *zài* being accepted in the literature as early as Li (1990). By adopting Talmy (2000b) notion of Motion event, the preposition *zài* does have a semantic function which is distinct from the one performed by the localizer. It says that the Figure is in a non-motion state, occupying a given position.

Building on the language-specific syntactical derivation by Wu (2015) and on the crosslinguistics observation by Landau and Jackendoff (1993), we also have proposed an alternative account to the claim according to which localizers turn common nouns into Place-word (Lamarre, 2007, among others). If we accept that localizers express the Axial Part, then it must also be assumed that they refer to the object as “where,” mentally represented via axes which allow us to identify its axially determined parts. This type of decomposition is entirely possible for nouns denoting things, but it is not viable with Place-words. It is, in fact, intuitive that a Place can be conceived as a deconstructable thing only if we consider it with reference to its component (20–22), or with reference to a position which requires a higher degree of specificity (24).

Based on this, we can reverse the generally accepted claim that the localizer turns a thing into a place. We can argue that the localizer can only be attached to a Ground that is mentally represented as a thing having an axial part (and hence, a *center*, a *top*, a *bottom*, etc.).

In conclusion to this paper, we can now outline a set of notions that might help to internalize the representation of spatial motion events in Chinese. Among them, as anticipated (ii) and (iv) are an original contribution outlined in this paper, whereas the others are extrapolated from the literature.

(i) To specify the exact position of the Figure, we need to mentally represent the Ground as something having axially determined parts (an object as “where”). Only in this way, a center, bottom, top, etc. can be identified. This mental representation is based on the identification of an Axial Part with reference to the Ground.
(ii) In Chinese locative structures, the Axial Part information is signaled by localizers. The Axial Part information can be specified only when axially determined parts can be identified. Thus, *the localizer does not turn a thing into a place*, but rather highlights a region in an “objectified where,” mentally represented via axes, consistently with Terzi (2010) definition of Place, and with Wu (2015) decomposition of Place into Ground and localizer.
(iii) The Axial Part information is underspecified only when the Ground is not considered as an axially determined object, i.e., with Place-nouns and stereotypical locations.
(iv) All Chinese spatial prepositions (*zài* included) encode the Path. More specifically, they denote the type of Motion events (Goal, Source, Locative, Route) related to the main predicate. Therefore, the *locative spatial function is encoded as a type of null-motion event*.

We can draw from this the following pedagogical implication. Learners can restructure their mental representation of spatial motion events, by fine-graining components that in English are conflated into the preposition. Therefore, the tripartite scheme used for prepositional phrases should be presented as a construction in which each component contributes to a different spatial meaning. Learners would then become familiar with the analycity of Chinese, which tends to mark explicitly all the semantic components involved in the mental representation of an event.

Localizer use must be related to a place conceived as an object that can be analyzed in terms of its axial part (and thus geometrical features), thus allows learners to understand when it can be omitted.

**FIGURE 1:**
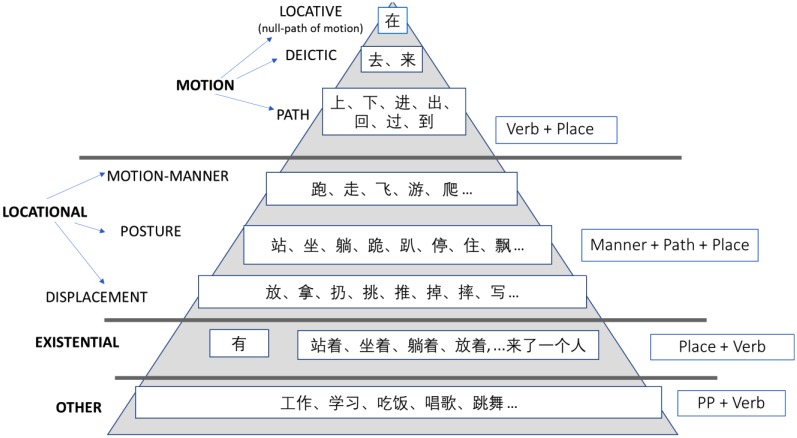
Verb Lexical-semantic classes.

Finally, other two notions have been pointed out that provides a rationale for understanding Chinese locative constructions. They are the *indefiniteness restriction effect* for explaining the canonical and locative inversion alternation, and the lexical semantic notion of locational verbs, which is essential for choosing between prepositional phrases and locative resultative constructions. A focus on the main lexical semantic categories is in order. The latter can be easily carried out through mental representation and diagraming (as Figure 1) aimed at helping learners “to identify main ideas, organize them into categories and reduce memory load,” in the spirit of Schraw (1998, p. 120) metacognitive strategies.

By internalizing the concepts above, learners might develop a representation of spatial motion events which is more consistent with the Chinese encoding of this type of content. At this stage, the full impact of this type of prescriptive knowledge in English–Chinese Interlanguage cannot be anticipated. Yet, this type of conceptual restructuring might enable adult learners to switch from L1 “thinking for speaking” to L2 “thinking for speaking” (Slobin, 1987).

## Author Contributions

The author confirms being the sole contributor of this work and has approved it for publication.

## Conflict of Interest Statement

The author declares that the research was conducted in the absence of any commercial or financial relationships that could be construed as a potential conflict of interest.

## Footnotes

1 For a survey on the Chinese and English typological variation (cf. Wu, 2014).

2 For a more extensive exegesis of the stationary (locative) type of Motion event (see Ma, 2016, p. 36ff).

3 Building on Nikitina (2008) and Lin (2013, p. 861) also proposes that localizers specify the “search domain,” that is, the “space anchored to the ground” where a physical object is located.

4 Cf. Lamarre (2007); the author provides several arguments for proving that path verbs in resultative compounds are undergoing a decategorialization (loss of ‘verbiness’). However, she also observes that they sometimes keep their verbal features; therefore, in the context of this paper, we categorize them as verbs.

5 For a general survey on the features of these sentences (see Sparvoli, 2016).

6 As demonstrated in a conspicuous literature, indefinites are allowed in subject position in environment such as in listing or when introduced by *yǒu*, for a survey on the topic, also with reference to Italian, see Sparvoli (2016) and Morbiato (2017).

## Abbreviations

BA: head of the BA construction
BEI: head of the passive construction
CL: classifier
DUR: durative aspect
PKU: Peking University Corpus: http://ccl.pku.edu.cn:8080/ccl_corpus/index.jsp?dir=xiandai
PRF: perfective aspect
SFP: sentence final particle
SUB: subordinator.
